# Prevalence and Clinical Correlates of Fibromyalgia Screening Positivity in Patients with Inflammatory Bowel Disease

**DOI:** 10.3390/jcm15031203

**Published:** 2026-02-03

**Authors:** Mohammad Mustafa, Yasser Bawazir, Mariam Mukhtar, Mahmoud Mosli, Nadeem Butt, Jana Jahhaf, Khalid Alghamdi, Roaa Alsolaimani

**Affiliations:** 1 Department of Internal Medicine, Faculty of Medicine, University of Jeddah, Jeddah 23218, Saudi Arabia; 2 Department of Internal Medicine, Faculty of Medicine, King Abdulaziz University, Jeddah 22254, Saudi Arabia; ymbawazir@kau.edu.sa (Y.B.); mmosli@kau.edu.sa (M.M.);; 3 Inflammatory Bowel Disease Research Group, King Abdulaziz University, Jeddah 22254, Saudi Arabia; 4 Department of Family and Community Medicine, Faculty of Medicine Rabigh, King Abdulaziz University, Jeddah 22254, Saudi Arabia; nshafique@kau.edu.sa; 5 Rheumatology Unit, King Khalid University Hospital, Riyadh 12372, Saudi Arabia; 6 School of Medicine, King Abdulaziz University, Jeddah 22254, Saudi Arabia

**Keywords:** fibromyalgia, inflammatory bowel disease, prevalence, predictors

## Abstract

**Background:** Inflammatory bowel disease (IBD) is associated with chronic pain and reduced quality of life, even in the absence of active intestinal inflammation. International studies suggest that fibromyalgia (FM), a chronic pain disorder characterized by widespread musculoskeletal pain, fatigue, sleep disturbance, and multiple somatic symptoms, is more prevalent among patients with IBD than among the general population. However, data from Saudi Arabia are limited. **Methods:** This cross-sectional study was conducted at King Abdulaziz University Hospital in Jeddah, Saudi Arabia, during July and August of 2024. Patients with biopsy-confirmed IBD were identified from hospital records and contacted by phone to screen for FM using a validated Arabic version of the Fibromyalgia Rapid Screening Tool. Demographic data, comorbidities, medication exposure, IBD characteristics, disease activity, and laboratory parameters were extracted from the medical records and compared between patients with and without FM. **Results:** Of 274 patients with IBD (mean age 30.9 ± 9.2 years; 56.9% male), 51 (18.6%; 95% CI 14.2–23.7) met criteria for FM. Patients with FM tended to be older than those without and were more likely to have comorbidities, particularly thyroid disorders, as well as low Vitamin D levels. Prior 5-aminosalicylic acid use was also more common among patients with FM. Inflammatory markers, hematological indices, IBD phenotypes, and disease activity were similar between the groups. **Conclusions:** Saudi patients with IBD often have comorbid FM. Routine FM screening in IBD clinics may help avoid misattributing central pain to active inflammation and unnecessary treatment escalation.

## 1. Introduction

Inflammatory bowel disease (IBD) is a chronic immune-mediated inflammatory disorder of the gastrointestinal tract. This condition follows a relapsing–remitting course and has heterogeneous clinical manifestations, such as Crohn’s disease (CD) and ulcerative colitis (UC), which differ in disease distribution but share overlapping pathogenic mechanisms and systemic features. Up to 40% of patients with IBD develop extraintestinal manifestations, most commonly involving the musculoskeletal system (approximately 17–39%), further impairing health-related quality of life [1,2,3].

Fibromyalgia (FM) is a chronic pain syndrome characterized by widespread musculoskeletal pain, fatigue, sleep disturbance, cognitive symptoms, and multiple somatic complaints. Increasingly recognized as a central sensitization disorder in which pain processing is amplified despite minimal peripheral tissue damage [4], FM may occur as a primary condition or coexist with chronic inflammatory and gastrointestinal diseases [1,4], FM affects approximately 2–3% of the global population, with a higher prevalence in women [4]. A recent systematic review and meta-analysis from Saudi Arabia estimated a pooled FM prevalence of 13.4% [5]. A cross-sectional study demonstrated that FM is common even in young Saudi populations, with a prevalence of 9.6% among medical students aged 18–26 years [6].

The clinical features of FM overlap substantially with those of IBD and numerous other conditions, particularly pain, fatigue, sleep disturbance, and impaired well-being, which results in frequent under recognition of FM in routine practice [1,4,5]. Genetic susceptibility, chronic nociceptive input, and psychosocial stressors are highly relevant factors in both FM and IBD [1,3].

Several studies have shown that FM and chronic widespread pain are more prevalent in patients with IBD than in the general population, with an FM prevalence of approximately 10–15% among patients with IBD. This relationship often occurs in the absence of parallel increases in objective inflammatory activity [7,8]. Emerging mechanistic studies support a role for central sensitization in a subset of patients with IBD-related pain [9,10].

Despite growing international evidence linking fibromyalgia to inflammatory bowel disease, most available data originate from Western populations, with limited representation from the Middle East. This gap is clinically relevant because Saudi Arabia has one of the highest reported background prevalences of fibromyalgia worldwide, as well as a young and rapidly growing IBD population with distinct demographic, genetic, and healthcare characteristics. Extrapolating findings from Western cohorts may therefore be inappropriate. To date, no study has systematically evaluated fibromyalgia prevalence and correlates in Saudi patients with IBD using a validated Arabic screening instrument. This study addresses this gap by providing the first region-specific prevalence estimates and by exploring demographic, clinical, and laboratory correlates of fibromyalgia in a Middle Eastern tertiary-care IBD population. Therefore, this study aimed to determine the prevalence of FM among Saudi patients with IBD and to identify associated demographic, clinical, and laboratory factors to support more accurate diagnosis and patient-centered care.

## 2. Materials and Methods

### 2.1. Study Design and Setting

This cross-sectional study was conducted at King Abdulaziz University Hospital (KAUH), a tertiary care center in Jeddah, Saudi Arabia, during July and August of 2024. Hospital records were used to identify patients aged 12 years or older with biopsy-verified IBD (CD or UC) who were being followed regularly in the IBD clinic at KAUH.

### 2.2. Participants and Data Collection

Eligible patients were contacted via telephone and invited to participate. A total of 312 eligible patients were identified from clinic records. Of these, 274 agreed to participate and completed FiRST screening (response rate 87.8%). After verbal consent was obtained, FM screening was performed and clinical data were retrieved from electronic medical records. Extracted variables included demographic characteristics (age, gender, body mass index and smoking status) and comorbidities (including diabetes, hypertension, thyroid disorders, connective tissue diseases and other chronic conditions). The IBD-related variables included diagnosis (CD vs. UC) and Montreal classification variables. For CD, these included age at diagnosis (A), disease location (L), disease behavior (B), and presence of perianal disease; for UC, extent (E) was recorded. In addition, UC disease activity was reported where available (as measured by the Mayo score). Medication history included exposure to 5-aminosalicylic acid (5-ASA), corticosteroid therapy (route of administration and response) and biologic therapy (ever vs. never exposed). Laboratory parameters (hemoglobin, iron indices including transferrin saturation, thyroid stimulating hormone [TSH], erythrocyte sedimentation rate [ESR], C-reactive protein [CRP] and serum vitamin D). Data were extracted by two investigators using a standardized form. Missing data were handled using complete-case analysis for each variable. Data extractors were not blinded to FiRST status.

### 2.3. Fibromyalgia Assessment

The validated Arabic version of the Fibromyalgia Rapid Screening Tool (FiRST) was utilized to screen for fibromyalgia [11]. FiRST is a concise self-report screening instrument consisting of six binary (yes/no) items aimed at identifying individuals with symptoms indicative of fibromyalgia, with a cutoff score of ≥5 considered a positive screen. In the Arabic validation study, FiRST demonstrated excellent diagnostic accuracy, with a sensitivity of 90% and a specificity of 85% when compared with established diagnostic criteria. As FiRST is a screening tool rather than a diagnostic instrument, individuals were categorized as FiRST-positive or FiRST-negative rather than diagnosed with confirmed fibromyalgia.

### 2.4. Statistical Analysis

Continuous variables were reported as either means with standard deviations (SD) or medians with interquartile ranges (IQR). Normality was evaluated using histograms and the Shapiro–Wilk test. Independent-samples *t*-tests and Mann–Whitney U tests were used for normal and non-normal distributions, respectively. Categorical variables were presented as frequencies and percentages. The chi-square test was used for comparisons, with Fisher’s exact test employed when expected cell counts were below 5. Statistical significance was defined as a two-sided *p*-value of less than 0.05. Analyses were performed using IBM SPSS Statistics for Windows (version 26.0; IBM Corp., Armonk, NY, USA).

Multivariable logistic regression analysis was performed to identify factors independently associated with FiRST-positive status. Variables included age, sex, comorbidity status, thyroid disorders, prior exposure to 5-aminosalicylic acid, and serum vitamin D level. Results are presented as odds ratios (ORs) with 95% confidence intervals (CIs).

## 3. Results

### 3.1. Baseline Characteristics

The patients studied (Table 1) comprised 274 patients with IBD, with a mean (standard deviation [SD]) age of 30.9 (9.2) years (range, 12–66 years); 56.9% (*n* = 156) were male. The mean (SD) body mass index (BMI) was 23.8 (6.0) kg/m^2^. Most patients were nonsmokers (86.4%, *n* = 236/273). Comorbidities were relatively uncommon, occurring in 15.4% (*n* = 39/253) of the patients, with a low prevalence of specific conditions, including diabetes (1.2%), hypertension (2.0%), thyroid disorders (1.6%), and connective tissue diseases (1.2%).

Regarding medication history (Table 2), a subset of patients (*n* = 134) had detailed data on corticosteroid administration, among whom the majority had received oral therapy (72.4%, *n* = 97). Most patients exposed to corticosteroids were corticosteroid-responsive (93.9%, *n* = 124/132). Over half of the patients studied (53.7%, *n* = 132/246) had a history of 5-ASA use. Among the 257 patients for whom biologic therapy data were available, 62.3% (*n* = 160) had been treated with at least one biological agent, while 37.7% (*n* = 97) had not received biologic therapy.

Laboratory parameters (Table 2) demonstrated a mean (SD) hemoglobin level of 12.6 (2.1) g/dL (*n* = 240). Inflammatory markers showed a mean (SD) ESR of 22.1 (22.6) mm/h (*n* = 188) and a mean (SD) CRP of 19.1 (37.6) mg/L (*n* = 217). The mean (SD) vitamin D level was 49.9 (36.7) nmol/L (*n* = 154), and 82.2% (*n* = 203/247) of the patients had transferrin saturation within the normal reference range. Data availability varied across the laboratory measures.

The IBD population (*n* = 274) predominantly had CD (64.0%) or UC (33.3%), with unclassified IBD (2.7%) comprising the remainder. Among those with CD, most were diagnosed between 17 and 40 years of age (67.5%), and the most common disease location was ileocolonic (61.0%), followed by ileal (26.2%) and colonic (12.8%). Disease behavior was predominantly inflammatory/non-stricturing (47.3%). Smaller proportions showed stricturing (29.1%) or penetrating (23.6%) disease, and 37.0% had perianal disease. In patients with UC, left-sided (41.4%) and extensive colitis (36.8%) were more common than proctitis (21.8%). Most patients with UC had moderate (50.6%) or severe (24.1%) disease, whereas fewer patients had mild disease (19.5%) or were in remission (5.7%).

### 3.2. Study Outcomes

Overall, 51 of 274 patients met FiRST criteria for FM, yielding a prevalence of 18.6% (95% CI 14.2–23.7). Patients with FM were significantly older than those without FM (median age 32.0 vs. 28.0 years; *p* = 0.012). The prevalence of comorbid conditions was higher in the patients who screened positive on FiRST than screening negative group (26.5% vs. 12.7%; *p* = 0.026). In particular, thyroid disorders were significantly more common among patients who screened positive on FiRST (6.5% vs. 0.5%; *p* = 0.021). No significant differences were found between the groups in sex distribution, BMI, smoking status, diabetes prevalence, hypertension, or connective tissue diseases.

A significantly higher proportion of patients who screened positive on FiRST had a history of 5-ASA use compared with those without (70.2% vs. 49.7%; *p* = 0.014). However, the proportion of patients receiving biologic therapy was similar between those with and those patients who screened negative on FiRST (66.7% vs. 61.3%; *p* = 0.615), suggesting that this treatment was not significantly associated with FM in the patients studied. No significant differences were found in corticosteroid administration or corticosteroid response patterns between the groups.

The most striking laboratory difference between the groups was observed for vitamin D: patients who screened positive on FiRST had significantly lower median serum vitamin D levels than those screened negative (29.4 nmol/L vs. 43.9 nmol/L; *p* = 0.004). No significant differences were found in TSH, inflammatory markers (ESR and CRP), or iron status (transferrin saturation) between patients who screened positive and those who screened negative for FiRST (Table 3).

No significant differences were found in the underlying IBD phenotype or disease activity between patients who screened positive and those who screened negative for FiRST (Table 4). The distribution of CD and UC was similar between the groups (*p* = 0.228). Among patients with CD, there were no significant differences in age at diagnosis (Montreal A), disease location (Montreal L, including upper gastrointestinal involvement), disease behavior (Montreal B), or perianal disease. Similarly, among patients with UC, no significant differences were observed in disease extent (Montreal E) or disease activity, as measured by the Mayo score. These findings indicated that the presence of FM was not associated with a more severe or specific IBD phenotype in the patients studied.

In multivariable logistic regression analysis (Table 5), prior use of 5-aminosalicylic acid (5-ASA) remained independently associated with FiRST-positive status (OR 2.86, 95% CI 1.08–7.59; *p* = 0.035). Serum vitamin D level showed a non-significant trend toward association with FiRST positivity (OR 0.98 per unit increase, 95% CI 0.96–1.00; *p* = 0.068). Age, sex, overall comorbidity burden, and thyroid disorders were not independently associated with FiRST-positive status.

## 4. Discussion

In this cross-sectional study of 274 patients with IBD at a Saudi tertiary care center, nearly one in five (18.6%) screened positive for FM using the validated Arabic FiRST tool. Patients positive for FM were older, had a higher overall comorbidity burden, a higher prevalence of thyroid disorders, and had significantly lower vitamin D levels than those negative for FM. In contrast, hemoglobin, iron indices, TSH, ESR, and CRP levels were similar between the groups. The patterns of corticosteroid and biologic therapy use did not differ substantially, although there was a higher proportion of prior 5-ASA exposure among patients who screened positive on FiRST. The results also demonstrated that FM status was not associated with the IBD phenotype, Montreal classification variables, or UC disease activity, which indicates that FM may represent a clinically relevant comorbidity rather than a marker of more severe intestinal disease.

Previous studies have consistently demonstrated that FM and chronic widespread pain are more prevalent in patients with IBD than in the general population. Moreover, population-based and cohort studies have established FM as part of the musculoskeletal and pain spectrum of IBD [9,10,12,13]. Registry-based studies have further demonstrated that IBD was associated with an increased risk of subsequently developing FM or chronic widespread pain [14,15,16,17,18,19,20,21]. More recent cohorts using contemporary FM criteria have linked this condition to greater pain, fatigue, psychological distress, and impaired quality of life, often without parallel increases in objective inflammatory activity [14,15,16]. Mechanistic studies support a model of central sensitization driven by chronic nociceptive input and psychosocial factors [20,21]. Our findings expand on this research and apply it to a population of Saudi patients with IBD and confirm the clinical relevance of FM in this regional context.

To the best of our knowledge, this study is the first to provide data on the screening prevalence of FM among Saudi patients with IBD using a validated Arabic screening tool. The observed FM prevalence of 18.6% among patients with IBD was substantially higher than the general population estimates and broadly consistent with international IBD cohorts, although slightly higher than most hospital-based reports [9,10,13,14]. Our data also exceeded the available Saudi general population estimates, supporting the concept that chronic inflammatory disease confers additional FM risk beyond the background prevalence [5,6,22]. Our findings further demonstrate the feasibility of brief FM screening in routine gastroenterology practice.

The finding that thyroid disorders were more frequent among patients who screened positive on FiRST has not been consistently reported in prior IBD–FM studies but aligns with the recognized coexistence of FM with other endocrine and somatic conditions [14,15,16,22]. This association is clinically relevant, as thyroid disorders may contribute to musculoskeletal symptoms such as diffuse myalgia and fatigue that overlap with fibromyalgia.

Our results were consistent with the existing literature showing no strong association between FM and specific IBD treatments [9,10,23]. The observed association with 5-ASA should be interpreted cautiously, as it is unlikely to reflect a direct drug effect and may instead represent confounding by disease duration or treatment escalation patterns. In particular, patients who received 5-ASA alone might represent a subgroup with long-standing or suboptimally controlled disease and more persistent pain, which could increase the likelihood of FM symptoms being present and recognized; this idea supports a non-causal and exploratory interpretation of this association.

Our findings on vitamin D levels, inflammatory markers, and iron indices were novel in the IBD–FM literature. They are clinically relevant because vitamin D deficiency is a recognized contributor to musculoskeletal pain and fatigue [23,24,25]. However, given the cross-sectional design and the absence of supplementation data, this association should be interpreted cautiously and may reflect confounding or reverse causality.

Our results regarding the relationship between FM and IBD phenotype, disease extent, and activity were consistent with previous reports showing that FM does not correspond to specific Montreal classifications or more aggressive diseases [14,15]. Instead, FM appears to represent a superimposed pain amplification syndrome that can confound symptom-based assessments of IBD activity [20,21]. Distinguishing FM-related symptoms from inflammatory disease is therefore essential to avoid unnecessary treatment escalation [23,24,25].

Notably, in multivariable analysis adjusting for age, sex, comorbidities, thyroid disorders, and vitamin D level, prior exposure to 5-ASA remained independently associated with FiRST positivity, whereas vitamin D level showed only a non-significant trend.

Clinically, these findings emphasize that persistent pain in patients with IBD should not automatically trigger treatment escalation, but should instead prompt evaluation for centralized pain syndromes, vitamin D deficiency, and psychosocial contributors. Incorporating brief fibromyalgia screening tools into routine IBD clinics may therefore improve diagnostic accuracy and reduce unnecessary exposure to immunosuppressive therapies.

This study had several limitations. First, the FiRST is a screening tool rather than a diagnostic instrument, and systematic rheumatological assessments were not performed to exclude IBD-associated spondyloarthritis; therefore, some patients with inflammatory musculoskeletal disease may have been misclassified as FiRST-positive. Second, the single-center, cross-sectional design limited causal inference and generalizability. Moreover, we did not evaluate several comorbidities and lifestyle factors known to be linked with fibromyalgia, such as depression, anxiety, sleep quality, physical activity, dietary habits, socioeconomic status, and family history, which might have affected symptom reporting and the associations we observed. In addition, several IBD-related parameters were not assessed, including disease duration, longitudinal disease severity, and the direct impact of fibromyalgia on IBD management outcomes such as treatment escalation, healthcare utilization, or hospitalization rates. These variables are highly relevant but typically require prospective or longitudinal study designs; given the cross-sectional nature of the present study and reliance on available clinical records, we were unable to reliably capture these dimensions. Finally, patient-reported outcomes such as fatigue and quality of life were not assessed.

## 5. Conclusions

This study showed that FM is a common comorbidity among patients with IBD at KAUH. Of 274 patients with IBD, 18.6% were affected by FM, a prevalence higher than that reported in the general population. Although FM was not associated with the IBD phenotype, disease extent, or inflammatory markers, it was linked to older age, greater comorbidity burden, hypothyroidism, and lower vitamin D levels. These findings support FM as a source of pain and symptom amplification rather than active intestinal inflammation. Routine FM screening using validated tools may help guide appropriate, multidisciplinary, and patient-centered management, and avoid unnecessary escalation of IBD therapy. Future prospective studies incorporating standardized rheumatologic evaluation and longitudinal follow-up are needed to better delineate FM and other musculoskeletal manifestations of IBD.

## Figures and Tables

**Table 1:** Demographic and Clinical Characteristics of the patients studied (*n* = 274).

Variable	Overall (*n* = 274)
**Age, years**	
Mean (SD)	30.9 (9.2)
Range	12.0–66.0
**Gender**	
Female	118 (43.1%)
Male	156 (56.9%)
**Anthropometrics**	
Weight, Kg; Mean (SD) [Range]	64.4 (19.6) [28.0–167.0]
Height, cm; Mean (SD) [Range]	163.3 (11.4) [102.0–190.0]
BMI, kg/m^2^; Mean (SD) [Range] (*n* = 273)	23.8 (6.0) [10.9–52.8]
**Smoking Status** (*n* = 273)	
Smoker	28 (10.3%)
Ex-smoker	9 (3.3%)
Non-smoker	236 (86.4%)
**Comorbidities** (*n* = 253)	
Any Comorbidity	39 (15.4%)
No Comorbidity	214 (84.6%)
Diabetes (*n* = 248)	3 (1.2%)
Hypertension (*n* = 248)	5 (2.0%)
Thyroid Disorder (*n* = 248)	4 (1.6%)
Connective Tissue Disease (*n* = 248)	3 (1.2%)

Note: Percentages are based on available data; denominators vary due to missing values.

**Table 2:** Medication History and Laboratory Parameters of the patients studied (*n* = 274).

Variable	Overall (*n* = 274)
**Corticosteroid Administration** (*n* = 134)	
Oral (P.O.)	97 (72.4%)
Intravenous (I.V.)	2 (1.5%)
Both (P.O. and I.V.)	35 (26.1%)
**Corticosteroid Response** (*n* = 132)	
Corticosteroid-responsive	124 (93.9%)
Corticosteroid-dependent course	8 (6.1%)
**5-ASA Use** (*n* = 246)	
Ever	132 (53.7%)
Never	114 (46.3%)
**Biologic therapy use (** ***n*** ** = 257)**	
Ever	160 (62.3%)
Never	97 (37.7%)
**Laboratory Values**	
Hemoglobin, g/dL; Mean (SD) [Range] (*n* = 240)	12.6 (2.1) [7.1–17.5]
Platelet Distribution Width (PDW), %; Mean (SD) [Range] (*n* = 226)	11.8 (2.2) [6.8–19.7]
Ferritin, ng/mL; Mean (SD) [Range] (*n* = 179)	63.0 (132.4) [0.4–1549.0]
TSH, µIU/mL; Mean (SD) [Range] (*n* = 63)	2.7 (3.6) [0.3–29.0]
Erythrocyte Sedimentation Rate (ESR), mm/hr; Mean (SD) [Range] (*n* = 188)	22.1 (22.6) [0.0–115.0]
C-Reactive Protein (CRP), mg/L; Mean (SD) [Range] (*n* = 217)	19.1 (37.6) [2.9–290.0]
Vitamin D, nmol/L; Mean (SD) [Range] (*n* = 154)	49.9 (36.7) [7.5–287.0]
**Transferrin Saturation** (*n* = 247)	
Normal	203 (82.2%)
Low	42 (17.0%)
High	2 (0.8%)

**Table 3:** Medication History and Laboratory Parameters of IBD Patients with and without Fibromyalgia.

Variable	FiRST-Positive (*n* = 51)	FiRST-Negative (*n* = 223)	Total (*n* = 274)	*p*-Value
**Corticosteroid Administration, *n*** ** (%)**				0.208
Oral (P.O.)	24 (85.7)	73 (68.9)	97 (72.4)	
Intravenous (I.V.)	0 (0.0)	2 (1.9)	2 (1.5)	
Both (P.O. and I.V.)	4 (14.3)	31 (29.2)	35 (26.1)	
**Corticosteroid Response, ** ***n*** ** (%)**				1.000
Corticosteroid-dependent course	1 (3.6)	7 (6.7)	8 (6.1)	
Corticosteroid-responsive	27 (96.4)	97 (93.3)	124 (93.9)	
**5-ASA Use, ** ***n*** ** (%)**				**0.014**
Ever	**33 (70.2)**	**99 (49.7)**	**132 (53.7)**	
Never	14 (29.8)	100 (50.3)	114 (46.3)	
**Biologic Therapy Use, ** ***n*** ** (%)**				0.615
Ever	30 (66.7%)	130 (61.3%)	160 (62.3%)	
Never	15 (33.3%)	82 (38.7%)	97 (37.7%)	
**Laboratory Values; Median (IQR)**				
TSH, µIU/mL	2.7 (1.6 to 3.5)	1.8 (1.2 to 2.8)	2.1 (1.3 to 3.1)	0.097
ESR, mm/hr	17.0 (10.2 to 22.8)	14.0 (5.0 to 32.8)	14.5 (5.8 to 32.0)	0.609
CRP, mg/L	3.3 (3.2 to 15.8)	3.8 (3.2 to 14.4)	3.5 (3.2 to 15.2)	0.605
**Vitamin D level, nmol/L**	**29.4 (20.3 to 41.2)**	**43.9 (28.2 to 68.6)**	**41.0 (26.0 to 64.2)**	**0.004**
**Transferrin Saturation, *n*** ** (%)**				0.890
Normal	39 (81.2)	164 (82.4)	203 (82.2)	
Low	9 (18.8)	33 (16.6)	42 (17.0)	
High	0 (0.0)	2 (1.0)	2 (0.8)	

Note: Percentages are based on available data; denominators vary due to missing values.

**Table 4:** IBD Phenotype and Activity According to Fibromyalgia Status.

Variable	FiRST-Positive (*n* = 51)	FiRST-Negative (*n* = 223)	Total (*n* = 274)	*p*-Value
**Diagnosis, ** ***n*** ** (%)**				0.228
Ulcerative Colitis	21 (42.0)	66 (31.3)	87 (33.3)	
Crohn’s Disease	29 (58.0)	138 (65.4)	167 (64.0)	
IBD-U	0 (0.0)	7 (3.3)	7 (2.7)	
**CD: Age at Diagnosis (Montreal A), ** ***n*** ** (%)**	(*n* = 29)	(*n* = 137)	(*n* = 166)	0.221
A1 (<16 years)	9 (31.0)	41 (29.9)	50 (30.1)	
A2 (17–40 years)	18 (62.1)	94 (68.6)	112 (67.5)	
A3 (>40 years)	2 (6.9)	2 (1.5)	4 (2.4)	
**CD: Location (Montreal L), *n* (%)**	(*n* = 29)	(*n* = 135)	(*n* = 164)	0.553
L1 (Ileal)	7 (24.1)	36 (26.7)	43 (26.2)	
L2 (Colonic)	2 (6.9)	19 (14.1)	21 (12.8)	
L3 (Ileocolonic)	20 (69.0)	80 (59.3)	100 (61.0)	
L4 (Upper GI)	3 (10.3)	8 (6.2)	11 (6.9)	0.617
**CD: Behavior (Montreal B), ** ***n *** **(%)**	(*n* = 29)	(*n* = 136)	(*n* = 165)	0.749
B1 (Non-stricturing, non-penetrating)	12 (41.4)	66 (48.5)	78 (47.3)	
B2 (Stricturing)	9 (31.0)	39 (28.7)	48 (29.1)	
B3 (Penetrating)	8 (27.6)	31 (22.8)	39 (23.6)	
**CD: Perianal Disease, ** ***n*** ** (%)**	(*n* = 28)	(*n* = 134)	(*n* = 162)	0.831
Yes	11 (39.3)	49 (36.6)	60 (37.0)	
No	17 (60.7)	85 (63.4)	102 (63.0)	
**UC: Disease Location (Montreal E), ** ***n*** ** (%)**	(*n* = 21)	(*n* = 66)	(*n* = 87)	0.197
E1 (Proctitis)	7 (33.3)	12 (18.2)	19 (21.8)	
E2 (Left-sided)	9 (42.9)	27 (40.9)	36 (41.4)	
E3 (Extensive)	5 (23.8)	27 (40.9)	32 (36.8)	
**UC: Severity (Mayo Score), ** ***n *(%)**	(*n* = 21)	(*n* = 66)	(*n* = 87)	0.718
S0 (Remission)	1 (4.8)	4 (6.1)	5 (5.7)	
S1 (Mild)	6 (28.6)	11 (16.7)	17 (19.5)	
S2 (Moderate)	10 (47.6)	34 (51.5)	44 (50.6)	
S3 (Severe)	4 (19.0)	17 (25.8)	21 (24.1)	

**Table 5:** Multivariable Logistic Regression Analysis of Factors Associated with FiRST-Positive Status.

Variable	Coefficient (B)	SE	OR	95% CI	*p*-Value
Intercept	−1.183	0.982	N/A	N/A	0.2285
Age	0.000	0.025	1.00	[0.95, 1.05]	0.9988
Gender (Male vs. Female)	−0.388	0.475	0.68	[0.27, 1.72]	0.4136
Comorbidity (Yes vs. No)	0.384	0.639	1.47	[0.42, 5.13]	0.5474
Thyroid Disorder (Yes vs. No)	0.996	1.377	2.71	[0.18, 40.26]	0.4698
5-ASA (Ever vs. Never)	1.050	0.498	2.86	[1.08, 7.59]	0.0351
Vitamin D level	−0.018	0.010	0.98	[0.96, 1.00]	0.0680

Note. B = logistic regression coefficient; OR = odds ratio; SE = standard error; CI = confidence interval. Statistical significance was set at *p* < 0.05.

## Data Availability

Due to confidentiality agreements, the raw data utilized in this research cannot be disclosed. Nonetheless, summary statistics or further information can be provided upon reasonable request, pending approval from the appropriate authorities.
